# Sepsis Alerts in Emergency Departments: A Systematic Review of Accuracy and Quality Measure Impact

**DOI:** 10.5811/westjem.2020.5.46010

**Published:** 2020-08-24

**Authors:** Matthew I. Hwang, William F. Bond, Emilie S. Powell

**Affiliations:** * University of Illinois College of Medicine at Peoria, Peoria, Illinois; † University of Illinois College of Medicine at Peoria, OSF HealthCare, Jump Simulation and Department of Emergency Medicine, Peoria, Illinois; ‡ Northwestern University Feinberg School of Medicine, Northwestern Memorial Hospital, Department of Emergency Medicine, Chicago, Illinois

## Abstract

**Introduction:**

For early detection of sepsis, automated systems within the electronic health record have evolved to alert emergency department (ED) personnel to the possibility of sepsis, and in some cases link them to suggested care pathways. We conducted a systematic review of automated sepsis-alert detection systems in the ED.

**Methods:**

We searched multiple health literature databases from the earliest available dates to August 2018. Articles were screened based on abstract, again via manuscript, and further narrowed with set inclusion criteria: 1) adult patients in the ED diagnosed with sepsis, severe sepsis, or septic shock; 2) an electronic system that alerts a healthcare provider of sepsis in real or near-real time; and 3) measures of diagnostic accuracy or quality of sepsis alerts. The final, detailed review was guided by QUADAS-2 and GRADE criteria. We tracked all articles using an online tool (Covidence), and the review was registered with PROSPERO registry of reviews. A two-author consensus was reached at the article choice stage and final review stage. Due to the variation in alert criteria and methods of sepsis diagnosis confirmation, the data were not combined for meta-analysis.

**Results:**

We screened 693 articles by title and abstract and 20 by full text; we then selected 10 for the study. The articles were published between 2009–2018. Two studies had algorithm-based alert systems, while eight had rule-based alert systems. All systems used different criteria based on systemic inflammatory response syndrome (SIRS) to define sepsis. Sensitivities ranged from 10–100%, specificities from 78–99%, and positive predictive value from 5.8–54%. Negative predictive value was consistently high at 99–100%. Studies showed some evidence for improved process-of-care markers, including improved time to antibiotics. Length of stay improved in two studies. One low quality study showed improved mortality.

**Conclusion:**

The limited evidence available suggests that sepsis alerts in the ED setting can be set to high sensitivity. No high-quality studies showed a difference in mortality, but evidence exists for improvements in process of care. Significant further work is needed to understand the consequences of alert fatigue and sensitivity set points.

## INTRODUCTION

Sepsis is defined as life-threatening organ dysfunction due to a dysregulated inflammatory response to infection.^1^ It is implicated in an estimated 1.7 million hospitalizations each year and is among the most costly conditions for hospitals.^2^,^3^ Delays in diagnosis of sepsis can lead to delay in treatment,^4^,^5^ which can lead to increased morbidity and mortality.^6^ Quality measures now track time to these treatments as process markers of successful care.^7^ While studies have questioned some of the interventions, such as protocol-driven fluid resuscitation,^8^ there is general agreement that early antibiotic administration reduces mortality from sepsis.^6^,^9^–^11^

Risk for delays in diagnosis led to the development of automatic electronic sepsis alerts built into electronic health record (EHR) systems.^10^,^12^,^13^ Some of these systems were created for use in the inpatient ward,^14^,^15^ intensive care unit (ICU),^16^,^17^ and emergency department (ED),^18^,^19^ and some stretch across settings within a healthcare system.^20^,^21^ One study demonstrated that over 75% of sepsis hospitalizations presented in the ED, warranting a focused study of this population.^22^

The challenge of demonstrating the marginal impact of these systems is that they act alongside existing sepsis care processes in a very ill population whose incremental change in mortality may be difficult to detect. In addition, thanks to education campaigns for staff,^10^ the drive toward improvement in quality measures,^23^ and increasing board certification of emergency providers,^24^ ED personnel have become better trained and are likely better at detecting sepsis. Thus, in the highly visually and electronically monitored ED setting, the benefit of these systems over clinician gestalt may diminish over time.

The possibility still exists that automated sepsis alerts may be an important method to detect more subtle cases or earlier presentations and may have greater value in less monitored settings. The value of these alert systems is measured based on their detection accuracy, with a goal of high sensitivity and, more importantly, their impact on process or outcome measures. However, alert systems carry a risk of alarm fatigue and distraction.^25^,^26^ Sepsis alerts add to already increasing alarms with the EHR, including those for physiology monitors, pharmacy checking, and infectious disease isolation. The positive impact of these automated sepsis alerts and their alarm methods on sepsis care, specific to the ED, remains an open question, and drove the desire for this systematic review.

Alert systems vary in their criteria. Early systems were often rule-based using the Centers for Medicare and Medicaid Services (CMS) Sepsis-1 definition of sepsis: two of four systemic inflammatory response syndrome (SIRS) criteria with a suspected or identified infection source. SIRS is defined as at least two of the following four findings: temperature >38° Celsius (C) (100.4° Fahrenheit [F]) or <36°C (96.8°F); heart rate >90 beats/minute; respiratory rate >20 breaths/minute; or white blood count >12,000 per microliter (μL) or <4000/μL or 10% band forms.^1^ CMS with sepsis-2 set elevated temperature at >38.3°C (100.9°F).^27^ More advanced systems are using algorithms, which expand on the limited criteria of rule-based systems. Such criteria may include past medical history and lab values or vitals with near-real time updating.

Evaluation of the success of these systems is complicated by difficulty establishing consensus^28^ and evolving definitions for the sepsis spectrum, including the 2016 update to sepsis-3.^1^ Thus, the diagnostic criteria are both evolving and in most cases based on discharge diagnosis, rather than information available in the ED. The ability to accurately diagnose and treat a specific disease may be measured by studying discharge diagnosis, but it may not account for clinician decisions made with limited information, as is often encountered in ED settings. Discharge diagnosis as a standard does not account for a clinician’s ability to risk stratify and exclude life- threatening conditions, which is valuable for stabilizing patients and completing the diagnostic workup. Although using chief complaint for quality evaluation or diagnostic criteria has been proposed, it has yet to be standardized.^29^,^30^

**Population Health Research Capsule**

What do we already know about this issue?

The use of automated clinical alerts is increasing, and complex algorithmic models are now being implemented.

What was the research question?

How do sepsis alert systems in the emergency department perform based on accuracy and quality measures?

What was the major finding of the study?

Process measures moderately improved. One low-quality study showed mortality benefit, while no high-quality studies did.

How does this improve population health?

Further research of alert system elements is needed. Our goal is to guide the development of sepsis alerts to improve outcome measures.

Due to evolving systems and definitions, we systematically reviewed studies assessing the effectiveness of these alerts. Our objectives were to determine whether automated electronic sepsis alerts in the ED are accurate and whether they have an impact on quality measures and/or mortality.

## METHODS

This review followed guidelines presented by the Preferred Reporting Items for a Systematic Review and Meta-analysis of Diagnostic Test Accuracy Studies (PRISMA-DTA) and PRISMA-P.^31^,^32^ This review was registered with PROSPERO (Prospective Register of *Systematic Reviews)*.

### Search Strategy

Databases for the search included PubMed MEDLINE, Embase, the Cochrane Library, and the Cumulative Index of Nursing and Allied Health Literature (CINAHL), from the earliest available dates to August 2, 2018. We defined the search according to four fields: emergency department; sepsis; electronic health record; and alerts/alarms. Details of the search strategy are described in Appendix A.

### Eligibility Criteria

Randomized trials, performance improvement trials (including before and after studies), and cohort studies were included in the screening. Eligible studies included published articles with the following: 1) adult patients in the ED, diagnosed with sepsis, severe sepsis, or septic shock (hereafter referred to as sepsis); 2) an electronic system that alerts a healthcare provider of sepsis in real or near-real time; and 3) measures of diagnostic accuracy or impact on quality of care measures. Exclusion criteria included the following: 1) primary data based on non-ED settings, such as prehospital, ICU, or the general wards; 2) articles studying medical conditions that can present with sepsis, such as specific infections (eg, influenza), pregnancy-related issues, and bacteremia, without assessing sepsis independently; 3) alert systems that screen only at triage, as opposed to reaching an alert trigger threshold at any point in the ED visit; and 4) non-English language articles lacking translation. We ensured chosen articles came from peer-reviewed sources based on the presence of a peer-review process description on the journal homepage.

### Study Records

We collected citations in a reference manager software Zotero (Corporation of Digital Scholarship, George Mason University, Fairfax, VA). Article screening was completed through the online software Covidence systematic review software (Veritas Health Innovation, Melbourne, Australia). Two independent reviewers (authors WB and MH), selected for articles based on the inclusion and exclusion criteria in the title and abstract screenings. At the next stage, two independent reviewers (authors WB and EP) selected articles in the full-text screening. Conflicts were resolved through regular meetings or conference calls. Data was collected by WB and MH, and then extracted with Covidence to be stored as a secure Microsoft Excel file (Microsoft Corporation, Redmond, WA).

### Data Items

Qualitative data items for extraction included clinical setting, study design, age group, type of alert system, definition/threshold for the alert, method of alert notification, treatment recommendation, and reference standard. The implemented alert system was considered the index test. We classified the alert systems as rule based or algorithm based. Among the eligible studies, the rule-based alerts used SIRS criteria. The algorithmic alerts had unique measures such as vitals, Glasgow Coma Scale, and creatinine. Variations for either system are described in Table 1. Quantitative data items included sample size, population size, accuracy, and outcome measures.

### Outcomes and Prioritization and Diagnostic Accuracy Measures

We extracted data from articles on sepsis alerts for both diagnostic accuracy and impact on quality measures. Diagnostic accuracy assesses the ability of the alert to accurately detect sepsis. Measurements included positive and negative predictive values, sensitivity, and specificity. Quality measures of interest were process and outcome measures. Examples of process markers included compliance or time to antibiotic administration, fluid resuscitation, and lactate measurement. Outcome measures included mortality and length of ICU stay, although various additional markers were captured by different authors. When reported by the authors, we used confidence intervals for the given estimates.

### Data Synthesis

A qualitative analysis of each study was used. The variation of sepsis definition for the alerts, the set points, methods of alerting, response processes, etc prevented an aggregated quantitative analysis.

### Bias and Applicability

Covidence included a bias rating system based on the Cochrane standard of quality assessment. We added criteria from the Quality Assessment of Diagnostic Accuracy Studies 2 (QUADAS-2) to effectively assess diagnostic accuracy of the articles, per the recommendation of PRISMA-DTA, Leeflang, and Cochrane.^31^,^33^,^34^ We rated quality measure articles following the guidance of GRADE (Grades of Recommendation Assessment, Development and Evaluation).^35^ Each article was rated for bias regarding blinding of participants and personnel to the alert, blinding of outcome assessors, incomplete outcome data, selective outcome reporting, the index test, gold standard, and flow and timing. Once each component was finalized, a consensus overall quality rating was decided based on the risk of biases. The overall quality was scaled relative to the cohort study design. No articles had strong experimental designs (ie, randomized controlled trials); therefore, quality was ranked based on comparison within this cohort of articles. Details are recorded in Appendix B.

## RESULTS

### Study Selection and Characteristics

We imported 731 articles into Covidence. After duplicate removal, 693 were screened by title and abstract. Twenty articles underwent full-text assessment, and 10 were selected for the study (Figure).

Eight of these studies assessed diagnostic accuracy and six assessed quality measures. All studies were prospective or retrospective cohorts and were conducted in urban, tertiary and/or academic medical centers (Table 1). Publishing years ranged from 2009–2018. Two studies had algorithm-based alert systems, while eight had rule sets. All systems used different criteria based on SIRS to define sepsis. There was significant variability in the criteria used for activation of the sepsis alert, the threshold definitions that activated the alert, the presence or absence of triggering links to care order sets, and the degree and type of interventions triggered by the alert. Likewise, there were variations in the diagnostic criteria standards against which the alerts were weighed, with most studies using chart review confirmation, while some used clinician confirmation. Only Nguyen et al had a control group of 300 randomly selected patients during a study period when the alert did not fire.^44^ All of the other articles were either prospective or retrospective cohort designs without control groups.

### Diagnostic Accuracy

Diagnostic accuracy was recorded in Table 2 below. Specificity ranged from 78–99%, and positive predictive value (PPV) from 5.8% to 54%. Negative predictive value (NPV) was consistently high at 99–100%. Excluding Meurer et al,^41^ sensitivity ranged from 64–100%. Meurer et al had a sensitivity of 33.3% for the electronic alert alone, and 10.7% for the electronic alert and attending confirmation. With attending confirmation, specificity increased from 78.0% to 97.6%. The study had a low activation threshold of ≥2 SIRS criteria, the smallest sample size of 84, and an age range of 70 years or older. Patients were only included if they presented between 3 am and 9 pm on weekdays. This study also only included patients admitted from the ED, instead of all ED patients, risking selection bias. The notification system sent a page to the study coordinator before confirming with a physician.

In contrast, other studies directly notified a member of the clinical team, excluding Nguyen et al, which did not describe the notification method.^44^ Five rule-based studies were of high quality.^18^,^36^,^37^,^43^,^44^ Two studies had systems with high accuracy. Alsolamy et al^18^ had a sensitivity of 93.2%, specificity 98.4%, and PPV 21.0%. Bansal et al^37^ had a sensitivity of 100%, specificity 96.2%, and PPV 29.3%. Highest PPV was achieved by Nelson et al^43^ at 54.0% and Nguyen et al^44^ at 44.7%.

Austrian et al^36^ shared the number of total alerts fired, for any of three criteria sets including SIRS, nurse alert, and physician alert that included progressively more ill criteria. They report sensitivities of 73.0%, 23.8%, and 23.0%, respectively, and PPV of 13.0%, 22.4%, and 26.6% as expected for the more progressively stringent criteria. They did not share the denominator of all ED presenting patients for the retrospective period under study but report the total number of hospitalized sepsis patients based on discharge diagnosis. Septic patients may have been sent home, but if we assume they captured all true positives and false negatives through final diagnosis of sepsis, this allows for calculation of sensitivity and PPV and does not allow the calculation of specificity or NPV. With 2144 patients with a final diagnosis of severe sepsis or septic shock, and 97,216 alerts (any of the three levels included), they had the largest retrospective sample size.

Two studies assessing algorithm-based alerts were deemed low quality. Brown et al^39^ measured a sensitivity of 76.4%, specificity of 95.3%, and a low PPV of 5.8%. Martin Rico et al^40^ measured a sensitivity of 85%, specificity of 89%, and a PPV of 19%. Prevalence of sepsis compared to total ED patients was 0.3–2% in five studies.^18^,^37^,^39^,^40^,^43^ Meurer et al had a prevalence of 14.4%, but this was among patients ≥70 years old and it was the sole study with only SIRS criteria (a low threshold) for its sepsis definition.

### Quality Measures

Quality measures are described in Table 3. Two studies evaluating quality measures were high quality: Austrian et al^36^ and Nelson et al.^43^ In Austrian et al, process markers of time to first lactate and vasopressor use significantly improved. Statistically insignificant findings included blood cultures drawn before antibiotic administration and whether a lactate was drawn. Antibiotic timing was not reported. For Nelson et al, process markers of blood culture collection and chest radiograph before admission improved. Statistically insignificant findings included lactate collection and antibiotics given in the ED. Outcome measures of ICU transfer, ICU length of stay (LOS), and total LOS significantly improved for Austrian et al. Mortality did not improve significantly for either study.

Four studies (Bansal, Berger, Martin Rico, Narayanan)^37^,^38^,^40^,^42^ were judged to be of low quality. Berger et al had significant improvement in lactate testing. Narayanan et al improved antibiotics within 60 minutes, time to antibiotics, and LOS. Bansal et al^37^ had nearly 100% sensitivity and specificity with a modest PPV of 29.3%, and had no significant improvements in survival rate. Of note, we established the article to be high quality in regard to diagnostic accuracy, while outcome measures were low quality. In contrast, Austrian et al^36^ had improvements in LOS and Martin Rico et al^40^ had improvements in mortality, while both exhibited moderate accuracy.

None of the rule-based studies showed statistically significant improvements in mortality.^36^–^38^,^42^,^43^ The only outcome reported by an algorithm-based study (Martin Rico et al)^40^ was mortality, which showed significant improvement, although the study was judged to be of low quality. The alert system Narayanan et al^42^ studied did not recommend treatment as other systems did. For this rule-based system “antibiotics in 60 minutes” meant time to antibiotics, and LOS significantly improved.

## DISCUSSION

Overall, most of the study designs used to assess the impact of sepsis alerts were weak, and the review authors had difficulty isolating the impact of the automated sepsis alert itself from broader interventions such as response teams or order set bundles. Thus, our review conclusions must be couched within the strength of the overall low-quality evidence.

The limited evidence available suggests that sepsis alerts in the ED setting can be tuned to a high sensitivity for the detection of sepsis. Evidence from both low- and high-quality studies showed some improved process-of-care markers, including time to antibiotics, with the use of automated sepsis alerts.^36^,^38^,^40^,^42^,^43^ Lactate testing was studied by four groups with two producing significant results. Other than lactate measurement, no single measure consistently improved across studies. A lack of consistency of measured items and measurement methods creates a challenge in forming a conclusion. For example, one study examined whether blood cultures were collected, as opposed to blood cultures collected before antibiotic administration.

No high-quality studies showed a difference in mortality, and only one high-quality study showed impacts on ICU LOS and vasopressor use.^36^ Our findings are in keeping with a review by Makam in 2015 that covered alerts both inside and outside of the ED environment.^45^ Our review added recently published articles, including those that now use an algorithmic as opposed to simple rules-based approaches, and was focused on patients presenting to the ED. The strongest study designs we reviewed for inclusion were prospective cohort studies, but we would call attention to a well-executed performance improvement study conducted by Gatewood et al.^46^ They included a computerized alert with a multipronged intervention and showed a substantial improvement in sepsis bundle of care compliance. However, they did not show differences in mortality in part due to the inclusion of lower risk patients on the sepsis spectrum.

Sepsis alerts represent a difficult area to study with traditional randomized methods. One challenge is that in the course of operational improvement, sepsis alert criteria and/or alert thresholds may be subtly changed in the background. This may be done by information technology, analytics, or EHR personnel to address PPV or safety concerns, usually with a clinician’s input, but often without alerting all ED staff to the change. Moving to a more rigorous study design requires holding the alert constant and ethical approval for a non-alert or clinician gestalt arm. Thus, success will likely be found in future studies that use time series, or perhaps cluster randomized rollout methods across healthcare systems. Likewise, future areas for study could include comparisons of the method of alert, and the presence or absence of treatment recommendations.

None of the studies addressed potential harms. Harm may include the alarm issues impacting staff, missing alternative diagnoses due to early anchoring on sepsis, and the follow-on effects of early, aggressive fluid intervention, which has been questioned more broadly in the sepsis literature.^8^ Significant further work is needed on the alarm consequences of the sensitivity set points, and if possible, such work should incorporate influences from other non-sepsis alarms in alarm fatigue.

Although low quality, one algorithmic system showed significant mortality improvement, potentially validating its further development.^40^ Systems such as this are being developed to improve accuracy and PPV, and may include risk factors such as comorbid conditions and past medical history. These systems can effectively insert multiple variables into an equation using current and past patient data as regression coefficients, running the calculation repeatedly over the course of a patient stay as more predictor variables become available. The data creating the coefficients of such a regression-based equation would influence the predictor’s value. For example, a sepsis predictor tool based on the elderly would likely not be predictive for children. The newest models of sepsis alerts include machine learning. Complex algorithmic models may use well over 50 variables, and a machine-learning program may be integrated into them. Machine learning uses computer programming to identify patterns and significant predictors beyond the reasonable capabilities of humans. With continual analysis, it can fine-tune coefficients and thresholds of the algorithm. Initial studies show promise,^47^–^49^ and additional research is required to assess its impact on clinical outcomes.

## LIMITATIONS

Our limitations include a risk of publication bias because we did not search the gray literature or clinical trials for studies in progress. There are likely many hospital systems that have implemented sepsis alerts, collected data, and did not report it. Our consensus group was small in number, but we followed a rigorous process using review rubrics guided by well-accepted grading criteria.

## CONCLUSION

Automated sepsis alerts in the ED may be set to a high sensitivity. Process measures show moderate benefit; however, no single measure has consistently improved, and high-quality studies have yet to demonstrate, a mortality benefit. Specific components of these systems, alarm fatigue, and sensitivity set points should be examined further. Sepsis alerts demonstrate utility and future research is indicated to build a more ideal alert system.

## Supplementary Information





## Figures and Tables

**Figure:**
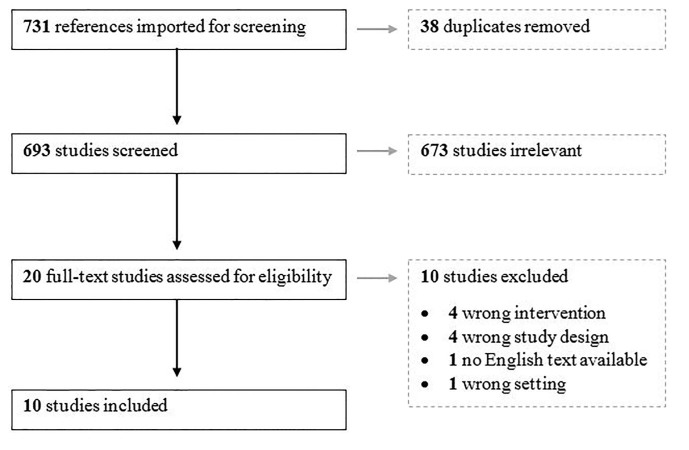
PRIMSA flow diagram.

**Table 1:** Characteristics of included studies.

Source (the article)	Study design	Demographic	Type of alert system (the index test)	Definition/Threshold for the alert	Method of alert notification	Treatment recommended	Reference standard (compared to the index test)
Alsolamy 2014^18^	Prospective cohort	>14 years old	Rule based	≥2 SIRS criteria and organ dysfunction, or 2 organ dysfunction criteria^	Notification to nurse who pages the physician	No	Clinical evaluation by an EM or ICU physician following 2012 surviving sepsis campaign guidelines
Austrian 2018^36^	Retrospective cohort	≥18 years old	Rule based	1st alert is SIRS based. 2nd and 3rd alerts are sepsis based, which is the SIRS alert plus systolic blood pressure <90mmHg or lactate ≥4 mg/dL	Electronic notifications to the following, Alert 1: nurse Alert 2: nurse Alert 3: provider	Yes (to all 3 alerts)	ICD-9 coding for severe sepsis or septic shock on admission only
Bansal 2018^37^	Prospective cohort	Adult patients (though not clearly specified)	Rule based	1st alert is SIRS based. 2nd alert is a sepsis alert, which is the SIRS alert plus WBC ≥12K or ≤4K Blood cultures ordered OR Lactate >4 mg/dL alone	Team leader paged	Yes, a sepsis response team in the post alert group	2 physician reviewers using standardized sepsis criteria, approved by Mayo Clinic enterprise subspecialty councils for EM and critical care
Berger 2010^38^	Prospective cohort	≥19 years old	Rule based	>2 SIRS criteria plus infection source	Electronic notification to clinician	Yes, lactate recommended	≥2 SIRS criteria and clinical suspicion, retrospectively
Brown 2016^39^	Prospective cohort	≥14 years old	Algorithm based	75 parameters including demographics, encounter details, lab tests, SIRS criteria, and other clinical measurements	Page and email to charge nurse	Not specified	Admitted from ED to ICU and either 1) ICD-9 discharge diagnosis relating to sepsis or infection or 2) identification by a QI coordinator in the ICU.
Martin Rico 2017^40^	Prospective cohort	≥14 years old	Algorithm based	Series of parameters including lab tests, SIRS criteria, vitals, and Glasgow coma scale score	Electronic notification to clinician	Yes, with an e-order set	Chart review with “clinical experts” with ICD-9 CM discharge diagnosis of sepsis
Meurer 2009^41^	Prospective cohort	≥70 years old	Rule based	≥2 SIRS criteria	Page to study coordinator who confirms a source of infection from the physician	No	Chart reviewers (3) confirmed or excluded the diagnosis
Narayanan 2016^42^	Retrospective cohort	≥18 years old	Rule based	1st alert is SIRS based. 2nd alert is a sepsis alert, which is the SIRS alert plus end organ dysfunction or fluid nonresponsive hypotension	Electronic notification to clinician	No	Chart review with ICD-9 code diagnosis of severe sepsis and septic shock
Nelson 2011^43^	Prospective cohort	≥18 years old	Rule based	≥2 SIRS criteria and 2 systolic blood pressure measurements less than 90mmHg	All caregivers notified with a page	Yes	Chart review with the same SIRS and hypotension criteria
Nguyen 2014^44^	Retrospective cohort	All ED patients*	Rule based	≥2 SIRS criteria, and systolic blood pressure ≤90mmHg or lactic acid ≥2.0mg/dL.	Not specified	Not specified	300 patients for which the alert did not fire were randomly selected

^ Systolic blood pressure <90 to 86 mmHg with intravenous fluids or <86 mm Hg regardless of fluids, blood oxygen saturation <90% to 85% with supplemental oxygen or <85% without oxygen, or lactate >2 mmol/L.

*SIRS*, systemic inflammatory response syndrome; *ICU*, intensive care unit; *ICD-9*, International Classification of Diseases, 9^th^ ed; *mmHG*, millimeters of mercury; *mg/dL*, milligram per deciliter; *mmol/L*, millimole per liter; *WBC*, white blood count; *K*, thousand; *EM*, emergency medicine; *ED*, emergency department; *QI*, quality improvement.

* ”While children have different ranges for SIRS criteria, <1% of emergency department (ED) patients were <18 years old…”

*SIRS*, systemic inflammatory response syndrome; *ICD-9*, International Classification of Diseases, 9^th^ ed; *mmHg*, millimeters of mercury; *mg/dL* milligram per deciliter; *ED*, emergency department.

**Table 2:** Diagnostic accuracy.

Source	Sample size (n)*	Population size (N)^	Sensitivity (95% CI)	Specificity (95% CI)	Positive predictive value (95% CI)	Negative predictive value (95% CI)	Overall Quality
Alsolamy 2014^18^	205	49,838	93.18 (88.78–96.00)	98.44 (98.33–98.55)	20.98 (18.50–23.70)	99.97 (99.95–99.98)	High
Austrian 2018^36^	1306	Not specified	73		14.6		High
Bansal 2018^37^	419	27106	100 (99.12–100)	96.21 (95.97–96.43)	29.3	100	High
Brown 2016^39^	352	93,733	76.4	95.3	5.8	99.9	Low
Martin Rico 2017^40^	178	37,323	85 (67.2–99.5)	89 (88.8–89.7)	19	99	Low
Meurer 2009^41^	Alert alone: 26 Alert and attending confirmation: 9	583	Alert alone: 33.3 (23.3–43.4) Alert and attending confirmation: 10.7 (4.1–17.3)	Alert alone: 78.0 (71.7–84.4) Alert and attending confirmation: 97.6 (95.2–99.9)			Low
Nelson 2011^43^	Sens. and Spec.: 1375 PPV and NPV: 1386	33460	64	99	54	99	High
Nguyen 2014^44^	795	Not specified			44.7 (41.2–48.2)		High

* Alerts for sepsis meeting the diagnostic criterion standard of the individual article.

^ Patients presenting to the emergency department (ED).

*CI*, confidence interval; *PPV*, positive predictive value; *NPV*, negative predictive value; *Sens. and Spec.*, sensitivity and specificity.

**Table 3:** Quality Measures.

Source	Sample size (n)*	Population size (N)^	Significant results (process/outcome marker: prior vs. after)	Insignificant results	Overall quality
Austrian 2018^36^	Before sepsis alert: 838 After Sepsis alert: 1306	2144#	ICU transfer: 36.9% vs. 25.8%, p<0.001 ICU length of stay (days): 1.8 (3.7) vs. 1.2 (3.1), p<0.001 Length of stay (days): 10.1 (SD 10.1) vs. 8.6 (SD 7.9), p<0.001 Time to first lactate (days): 0.19 (0.94) vs. 0.16 (0.58), p<0.001 Vasopressor used: 28.8% vs. 22.7%, p<0.01	Blood cultures drawn prior to antibiotics: 79.0% vs 79.2%, p=0.92 In-hospital mortality: 8.5% vs. 7%, p=0.22 Lactate drawn (excluding ≥24hrs after ED arrival): 90.7% vs. 91.3%, p=0.65	High
Bansal 2018^37^	Whole cohort: n=419 Severe sepsis and septic shock: n=252	27106		In-hospital survival rate with SSRT activation in full cohort: 0.69 (95% CI, 0.31 to 1.56) In-hospital survival rate with SSRT activation among severe sepsis/septic shock patients: 0.53 (95% CI, 0.26 to 1.11)	Low
Berger 2010^38^	Before sepsis alert: Lactate-151, Hyperlactatemia-33, Mortality-908. After sepsis alert: Lactate-366, Hyperlactatemia-54, Mortality-890	Before alert: 2903 After alert: 2893&	Lactate testing: 5.2% vs. 12.7% (95% CI, 6.0 to 9.0%) absolute increase p<0.001	Change in frequency of hyperlactatemia if lactate was tested: 21.9% vs. 14.8% (95% CI, −0.4 to 14.6) Mortality: 5.7% vs. 5.2% (95% CI, −1.6 to 2.6%, p=0.64)	Low
Martin Rico 2017^40^	1190	37,323	Mortality: 36.3% vs. 26.1% Adjusted risk reduction for mortality: 36% (0.43–0.97) Incidence Rate Ratio: 0.64, p=0.036		Low
Narayanan 2016^42^	Prior to sepsis alert: n=111 After sepsis alert: n=103	not specified	Antibiotics in 60 minutes: 48.6% vs. 76.7%, p<.001 Length of stay odds ratio: [0.66 (0.53–0.82)] Mean time to antibiotics (minutes): 61.5 (33–171) vs. 29 (2–59), p<.001	Mortality odds ratio: 0.64 (0.26–1.57)	Low
Nelson 2011^43^	184	33460	Blood culture collected odds ratio: [2.9 (1.1–7.7)] Chest radiograph before admission odds ratio: [3.2 (1.1–9.9)]	Antibiotic given in ED odds ratio: [2.8 (0.9–8.6)] Lactate collected odds ratio: [1.7 (0.9–3.2)]	High

* Alerts for sepsis meeting the diagnostic criterion standard of the individual article.

^ Patients presenting to the emergency department.

# All hospitalizations with severe sepsis or septic shock

& All patients with sepsis. Total ED presentations not specified.

*ICU*, intensive care unit; *vs*, versus; *SD*, standard deviation; *ED*, emergency department; *CI*, confidence interval; *SSRT*, sepsis and shock response team.
