# Supplementary material for: Sepsis Alerts in Emergency Departments: A Systematic Review of Accuracy and Quality Measure Impact
Source: West J Emerg Med. 2020 Aug 24;21(5):1201–10. doi: 10.5811/westjem.2020.5.46010 (PMC7514413; doi:10.5811/westjem.2020.5.46010)
Supplement: Supplementary file 1 [file wjem-21-1201-s001.docx]

**Appendix A. Search strategy**

Search date August 1^st^, 2018

**PubMed**

| Search | Query | Items found |
| --- | --- | --- |
| #5 | Search (#1 AND #2 AND #3 AND #4) | 293 |
| #4 | Search (Electronic Health Records[mh] OR health record* OR Medical Records Systems, Computerized[mh] OR medical record* OR Patient Records Systems OR information system OR electronic* OR computerized OR health information system OR informatics OR computer-assisted) | 2265271 |
| #3 | Search (alert OR alarm OR algorithm OR Early Diagnosis[mh] OR monitoring, physiologic OR advisory OR advisories OR "monitor alarm" OR automat* OR warning OR sniffer[tiab] OR screening[tiab] OR surveillance[tiab] OR recognition[tiab] OR prediction OR "decision support" OR "regression algorithm"[tiab] OR support vector machine OR machine learning OR artificial intelligence OR expert systems OR computer heuristics OR "early warning score") | 1834399 |
| #2 | Search ("Emergency department" OR "Emergency Medicine" OR "Emergency Service, Hospital"[mh] OR "Trauma Center" OR "Emergency Ward" OR "Emergency Unit" OR "Emergency Room") | 184037 |
| #1 | Search (sepsis[tiab] OR sepsis[majr] OR "septic shock"[tiab] OR "systemic inflammatory response syndrome"[tiab] OR SIRS[tiab]) | 146068 |

**EMBASE**

| Search | Query | Items found |
| --- | --- | --- |
| #5 | #1 AND #2 AND #3 AND #4 | 462 |
| #4 | 'electronic health record'/exp OR 'health record*' OR 'electronic medical record system'/exp OR 'medical record*' OR 'patient records systems' OR 'information system' OR electronic*:ab,ti OR computerized OR 'health information system' OR informatics OR 'computer assisted' | 1,529,958 |
| #3 | alert OR alarm OR algorithm OR 'Early Diagnosis'/exp OR 'physiologic monitoring' OR advisory OR advisories OR 'monitor alarm' OR automat* OR warning OR sniffer:ab,ti OR screening:ab,ti OR surveillance:ab,ti OR recognition:ab,ti OR prediction OR 'decision support' OR 'regression algorithm':ab,ti OR 'support vector machine' OR 'machine learning' OR 'artificial intelligence' OR 'expert systems' OR 'computer heuristics' OR 'early warning score' | 2,276,082 |
| #2 | 'Emergency department' OR 'Emergency Medicine' OR 'emergency health service'/exp OR 'Trauma Center' OR 'Emergency Ward' OR 'Emergency Unit' OR 'Emergency Room' | 349,419 |
| #1 | 'sepsis':ab,ti OR 'sepsis'/de OR 'septic shock':ab,ti OR 'systemic inflammatory response syndrome':ab,ti OR SIRS:ab,ti | 204,858 |

**Cochrane Library**

| Search | Query | Items found |
| --- | --- | --- |
| #1 | MeSH descriptor: [Sepsis] explode all trees | 4266 |
| #2 | "sepsis" or "septic shock" or "systemic inflammatory response syndrome" or SIRS:ti,ab,kw (Word variations have been searched) | 10087 |
| #3 | #1 or #2 | 11682 |
| #4 | MeSH descriptor: [Emergency Service, Hospital] explode all trees | 2500 |
| #5 | "Emergency department" or "Emergency Medicine" or "Trauma Center" or "Emergency Ward" or "Emergency Unit" or "Emergency Room" (Word variations have been searched) | 16214 |
| #6 | #4 or #5 | 16507 |
| #7 | MeSH descriptor: [Early Diagnosis] explode all trees | 1763 |
| #8 | sniffer or screening or surveillance or recognition:ti,ab,kw (Word variations have been searched) | 53433 |
| #9 | alert or alarm or algorithm or monitoring, physiologic or advisory or advisories or "monitor alarm" or automat* or warning or prediction or "decision support" or "regression algorithm" or support vector machine or machine learning or artificial intelligence or expert systems or computer heuristics or "early warning score" (Word variations have been searched) | 48933 |
| #10 | #7 or #8 or #9 | 97927 |
| #11 | MeSH descriptor: [Electronic Health Records] explode all trees | 319 |
| #12 | MeSH descriptor: [Medical Records Systems, Computerized] explode all trees | 639 |
| #13 | health record* or medical record* or Patient Records Systems or information system or electronic* or computerized or health information system or informatics or computer-assisted (Word variations have been searched) | 151506 |
| #14 | #11 and #12 and #13 | 151518 |
| #15 | #3 and #6 and #10 and #14 | 45 |

All results (45): Cochrane Reviews (22) Other Reviews (2) Trials (19) Methods Studies (0) Technology Assessments (1) Economic Evaluations (1) Cochrane Groups (0)

**CINAHL Plus with full text**

| S5 | S1 AND S2 AND S3 AND S4 | 45 |
| --- | --- | --- |
| S4 | MH "Electronic Health Records" OR health record* OR MH "Patient Record Systems" OR Patient Records Systems OR medical record* OR information system OR electronic* OR computerized OR health information system OR informatics OR computer-assisted | 223,234 |
| S3 | alert OR alarm OR algorithm OR MH Early Diagnosis OR monitoring, physiologic OR advisory OR advisories OR "monitor alarm" OR automat* OR warning OR TI sniffer OR AB sniffer OR TI screening OR AB screening OR TI surveillance OR AB surveillance OR TI recognition OR AB recognition OR prediction OR "decision support" OR TI "regression algorithm" OR AB "regression algorithm" OR support vector machine OR machine learning OR artificial intelligence OR expert systems OR computer heuristics OR "early warning score" | 316,457 |
| S2 | "Emergency department" OR "Emergency Medicine" OR MH "Emergency Service" OR "Trauma Center" OR "Emergency Ward" OR "Emergency Unit" OR "Emergency Room | 77,564 |
| S1 | TI sepsis OR AB sepsis OR MM "Sepsis" OR TI "septic shock" OR AB "septic shock" OR TI "Systemic Inflammatory Response Syndrome" OR AB "Systemic Inflammatory Response Syndrome" OR TI "SIRS" OR AB "SIRS" | 22,044 |
