# Supplementary material for: Sepsis Alerts in Emergency Departments: A Systematic Review of Accuracy and Quality Measure Impact
Source: West J Emerg Med. 2020 Aug 24;21(5):1201–10. doi: 10.5811/westjem.2020.5.46010 (PMC7514413; doi:10.5811/westjem.2020.5.46010)
Supplement: Supplementary file 2 [file wjem-21-1201-s002.docx]

**Appendix B. Quality assessment**

Assessment completed with the Covidence Software, which implements the Cochrane Risk of Bias Comparison. We used the GRADE rating system as a guide and added three measures of applicability per the QUADAS-2 rating system. Table 1 ranked low, high, or unclear levels of bias. Table 2 judged if there were concerns of applicability per QUADAS-2.

**Table 1.** Risk of Bias.

| Study | Alsolamy 2014 | Austrian 2018 | Bansal 2018 | Berger 2010 | Brown 2016 | Martin Rico 2017 | Meurer 2009 | Narayanan 2016 | Nelson 2011 | Nguyen 2014 |
| --- | --- | --- | --- | --- | --- | --- | --- | --- | --- | --- |
| Sequence Generation | Low | Low | Low | Low | High | Low | Low | Low | Low | Low |
| Blinding of participants and personnel for diagnostic accuracy | High | High | High | High | High | High | Low | Unclear^9^ | Low | Low |
| Blinding of participants and personnel for treatment | Low | Low | High | High | Low | Low | High | Low | Low | Low |
| Blinding of outcome assessors for diagnostic accuracy | Low | High | Low | High | High | Low | Unclear^8^ | Unclear^9^ | High | High |
| Incomplete outcome data for diagnostic accuracy | Unclear^1^ | Low | Low | Low | Low | Low | Low | Low | Low | Low |
| Incomplete outcome data for all outcomes | Low | Low | Low | Low | Low | Low | Low | Low | Low | Low |
| Selective outcome reporting | Low | Low | Low | Low | Low | Unclear^6^ | Low | Low | Low | Low |
| Index test | Low | Low | Low | Low | Low | Low | Low | Low | Low | Low |
| Flow and timing | Unclear^2^ | Low | High | Low | Low | Low | Low | Low | Low | Low |

**Table 2.** QUADAS-2 Applicability.

| QUADAS-2 applicability, patient selection | No | No | No | No | Unclear^4^ | No | Yes | No | No | No |
| --- | --- | --- | --- | --- | --- | --- | --- | --- | --- | --- |
| QUADAS-2 applicability, index test | No | No | No | No | No | No | No | No | No | No |
| QUADAS-2 applicability, reference standard | No | No | No | No | Unclear^5^ | No | Yes | No | No | Yes |

**Table 3.** Overall quality.

| Study | Alsolamy 2014 | Austrian 2018 | Bansal 2018 | Berger 2010 | Brown 2016 | Martin Rico 2017 | Meurer 2009 | Narayanan 2016 | Nelson 2011 | Nguyen 2014 |
| --- | --- | --- | --- | --- | --- | --- | --- | --- | --- | --- |
| Overall Quality | High | High | High/Low^3^ | Low | Low | Low^7^ | Low | Low | High | High |

^1^Unclear gold standard for sepsis.

^2^Timing of sepsis diagnosis is unclear

^3^Diagnostic accuracy was evaluated with high quality methods, while outcome measures were evaluated low quality methods.

^4^Some concerns because only ICU admitted ED patients were included, and they included 14-18 year old patients for an unknown reason.

^5^Additional identification by PI staff makes it unclear

^6^Sepsis with alert mortality is shown without sepsis mortality, which may potentially be selective reporting bias.

^7^No contemporaneous control group

^8^Does not explicitly state chart reviewers were used

^9^Unclear if reviewer was blinded
